# Diabetes burden and health expenditure in Türkiye and OECD countries: A value-based health care perspective

**DOI:** 10.12669/pjms.42.8.14019

**Published:** 2026-08

**Authors:** Ibrahim Halil Kayral, Demet Gokmen Kavak

**Affiliations:** 1 Ibrahim Halil Kayral Associate Professor, Izmir Bakırçay University, Izmir, Türkiye; 2 Demet Gokmen Kavak, PhD Turkish Medicines and Medical Devices Agency, Ankara, Türkiye

**Keywords:** Diabetes Mellitus, Health Expenditure, OECD, Türkiye, Value-Based Health Care

## Abstract

**Background & Objective:**

Diabetes mellitus is a major global cause of morbidity and mortality. In Türkiye, the diabetes burden has increased markedly, raising concerns about health system performance and expenditures. Our objective was to compare diabetes burden, health expenditures, and avoidable hospital admissions in Türkiye and Organisation for Economic Co-operation and Development (OECD) countries (2010-2021), and to examine their associations within a value-based health care (VBHC) framework.

**Methodology:**

This retrospective cross-national comparative study examined Türkiye and OECD countries between 2010 to 2021. Data were obtained from IHME-GBD-2021, OECD Health Statistics, and WHO National Health Accounts. Trend analyses and regression models were used to assess associations between diabetes-related disability-adjusted life years (DALYs), health expenditures, and financing indicators.

**Results:**

Türkiye’s diabetes-related DALY levels were consistently higher than the OECD average, with the gap widening from 18% in 2010 to 34% in 2021 (p=0.008). Preventable hospital admissions were also more frequent in Türkiye (242.7 vs. 147.9 per 100,000; p=0.006). Across OECD countries, higher per capita health expenditures showed negative associations with DALY rates, whereas in Türkiye rising expenditures were observed alongside higher DALYs. Out-of-pocket spending (OOPS) correlated positively with the diabetes burden in Türkiye, while a higher public share of expenditure (GGHE-D) was associated with reduced OOPS.

**Conclusion:**

Despite rising health expenditures, proportional improvements in diabetes outcomes have not been achieved in Türkiye. Strengthening primary care, preventive strategies, outcome measurement, and financial protection may support the transition toward value-based health care and help reduce the diabetes burden.

## INTRODUCTION

Diabetes mellitus (DM) is a leading global cause of illness and death. Global Burden of Disease (GBD) data identify it as a major contributor to disability-adjusted life years (DALYs), including years of life lost (YLL) and lived with disability (YLD).^1^ Its prevalence has risen sharply in low- and middle-income countries due to financial and system-level barriers.^2^

Although digital health tools such as e-Nabız and national lifestyle interventions may support prevention and monitoring, diabetes-related DALYs have continued to rise in Türkiye, suggesting that programme reach, uptake, or integration with routine chronic care may be insufficient to offset broader epidemiological trends.^3^-^5^ Rising expenditures alongside higher DALYs in Türkiye underscore efficiency gaps; strengthening primary care, prevention, and financial protection is essential for advancing VBHC.^6^ Regional studies link poor adherence, multimorbidity, and low physical activity to higher disease burden.^7^-^10^ Together, these findings stress the need for integrated prevention and patient-centred care.

Although per-capita health spending has increased, proportional improvements in diabetes outcomes have not been achieved, underscoring the need to link expenditures with measurable results.^11^ However, Türkiye’s performance has not yet been systematically compared with OECD peers from a VBHC perspective. This is the first national analysis linking Türkiye’s diabetes burden with financing indicators and value-based health metrics using OECD, WHO-GBD data. This study compares Türkiye and Organisation for Economic Co-operation and Development (OECD) countries (2010-2021) in diabetes burden, health spending, and preventable admissions, examining how financing structures affect outcomes. Linking DALYs with OOPS and GGHE-D, it evaluates whether higher spending creates patient value and offers policy insights for Türkiye and similar middle-income settings.

## METHODOLOGY

This cross-national comparative analysis used data from the GBD, OECD Health Statistics, and WHO Global Health Expenditure Database (2010-2021) to examine the links between diabetes outcomes, health spending, and financing structures.

### Ethical considerations:

As the study did not involve individual-level data, informed consent and ethical approval were not required.

### Data sources:

Diabetes burden indicators (DALY, YLL, YLD, mortality, prevalence) were derived from GBD; preventable hospital admissions (Avoidable Diabetes Hospital Admissions per 100,000 aged ≥15) from OECD Health Statistics; and expenditure indicators (OOPS, GGHE-D, CHE per capita) from WHO GHED. All data covered 2010-2021.

### Variables and measurement:

Dependent variables were the diabetes burden indicators, DALY and its components (YLD, YLL), and avoidable diabetes hospital admissions per 100,000 population aged ≥15. Independent variables included current health expenditure per capita (CHE, US$ PPP), CHE as a percentage of GDP, government health expenditure (GGHE-D, %CHE), and out-of-pocket spending (OOPS, %CHE). All monetary variables were adjusted for purchasing power parity, and disease burden indicators were standardized per 100,000 population following GBD, OECD, and WHO methodologies.

### VBHC framework:

This study used a macro-level VBHC-informed framework rather than evaluating provider-level VBHC implementation. In this framework, diabetes-related DALY, YLD, YLL, and avoidable diabetes hospital admissions represented health outcomes; CHE per capita represented spending level; and OOPS and GGHE-D represented financial protection and financing structure. Accordingly, value was interpreted as the extent to which health spending and financing arrangements were associated with lower diabetes burden, fewer avoidable admissions, and stronger financial protection.

### Hypotheses:

Based on international health economics frameworks and prior evidence, the study proposed seven hypotheses examining the relationships among diabetes burden, health expenditures, and financing structures in Türkiye and OECD countries.

**H1a.** Türkiye’s diabetes-related DALY rate is higher than the OECD average.

**H1b.** Türkiye’s avoidable diabetes hospital admission rate is higher than the OECD average.

**H1c.** In Türkiye, diabetes-related YLD is associated with avoidable diabetes hospital admissions.

**H2a.** In OECD countries, CHE per capita (US$ PPP) is inversely associated with diabetes-related DALY rates.

**H2b.** In Türkiye, CHE per capita (US$ PPP) is associated with diabetes-related DALY rates.

**H3a.** In Türkiye, OOPS (%CHE) is positively associated with diabetes-related DALY rates.

**H3b.** In OECD countries, GGHE-D (%CHE) is inversely associated with OOPS (%CHE).

Collectively, these hypotheses aimed to capture both comparative and structural dimensions of the diabetes burden within a value-based health care (VBHC) perspective.

### Statistical analysis:

Data preparation covered 2010-2021 continuous series. Only overlapping country-year pairs were used, excluding aggregate entries (e.g., “OECD-Total”). Analyses relied on complete cases without imputation. For Türkiye, avoidable diabetes admission data were limited to 2015-2017. Log transformations (e.g., ln (DALY), ln (CHE per capita)) ensured scale stability, and outliers were checked through time-series review and identifier harmonization. For H2a, an OECD panel fixed-effects model was used with country and year effects, and robust standard errors clustered by country. For H2b, a time-series OLS regression was used for Türkiye. In both models, DALY rates and CHE per capita were log-transformed.

The analysis followed four steps. First, annual trends (2010-2021) for diabetes burden (DALY, YLD, YLL), avoidable diabetes hospital admission, and expenditure indicators were examined, with means calculated for 2010-2013, 2014-2017, and 2018-2021. Second, Türkiye’s results were compared with OECD averages to test H1. Third, time-series analyses used Pearson and Spearman correlations, and when possible, OLS regressions to assess links among OOPS, CHE per capita, avoidable diabetes hospital admission, and DALY/YLD (H2b, H3a). For H1c (N=3), only descriptive Spearman results were reported. Fourth, a panel of OECD countries applied fixed-effects models with cluster-robust errors to test CHE-DALY (H2a) and GGHE-D-OOPS (H3b) associations. All analyses were performed using IBM SPSS Statistics version 27.

## RESULTS

Between 2010 and 2021, Türkiye’s diabetes-related DALY burden increased by approximately 56% based on the scaled DALY series used in the trend analysis, while the OECD average showed a more limited increase over the same period. Türkiye’s higher YLD values indicate greater quality-of-life losses, while YLL values remain lower but spiked during the pandemic. Overall, diabetes continues to impose a growing mortality and morbidity burden in Türkiye (IHME, 2024).

Türkiye-specific avoidable diabetes hospital admission data were available only for 2015-2017. During this period, Türkiye recorded 242.7 avoidable admissions per 100,000 population compared with an OECD mean of 147.9, and the difference was statistically significant (t = 12.75; p = 0.006). This finding suggests that diabetes-related avoidable admissions were higher in Türkiye during the available comparison period.^5^

In Türkiye, current health expenditure per capita (CHE per capita) increased by 155% from 2010-2021, rising from 742 US$ PPP to 1,827 US$ PPP. However, this increase runs counter to the rise in diabetes DALY and highlights the need to attend to spending-outcome efficiency. The OECD average over the same period increased from 3,250 to 5,120 US$ PPP (WHO, 2022; OECD, 2023). Türkiye’s total health expenditure as a share of GDP is 4.8%, while the OECD average is 9.2%. The out-of-pocket share (OOPS) in Türkiye stands at 16.4%, above the OECD average (13 -14%). This elevates the risk of inequities in access to care.^12^

Despite rising health expenditures, Türkiye’s growing diabetes burden has not declined, indicating inefficiency from a value-based health care (VBHC) standpoint. As Porter (2010) defines, value is the ratio of health outcomes to costs.^13^ The lack of outcome improvement despite higher spending suggests weak value generation.^14^,^15^

International evidence shows that in chronic diseases, spending growth alone is insufficient; systematic outcome measurement and stronger prevention are essential. OECD highlights that effective primary care and complication management improve cost-effectiveness and reduce avoidable hospitalizations.^5^

### H1a-H1c (Burden and hospital admissions):

**Fig.1:**
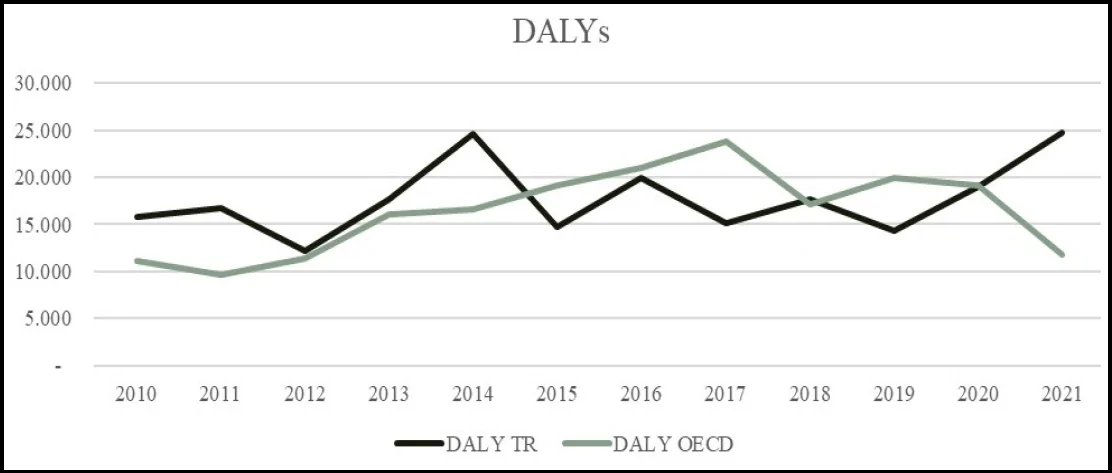
Diabetes-related DALY trends in Türkiye and OECD countries, 2010-2021. Data sources: IHME-GBD 2021; OECD Health Statistics 2023. Values represent the scaled DALY trend series used for comparative analysis.

Türkiye consistently exhibited higher diabetes-related DALY and hospital admission rates than the OECD average (p < 0.01), reflecting unmet preventive care needs (Fig.1). For 2015-2017, Türkiye recorded 242.7 avoidable admissions per 100,000 population compared with an OECD mean of 147.9 (p = 0.006). YLD and avoidable admission rates showed a positive descriptive pattern (ρ = 1.00, p = 0.046); however, given the very small number of Türkiye specific observations (N = 3), this finding is illustrative only and should not be interpreted as confirmatory evidence.

### H2a-H2b (Spending-outcome relationship):

Across OECD countries, higher per-capita health spending was significantly associated with lower diabetes DALY (β = -0.32, p < 0.001; Table-I). This supports H2a at the macro level. In Türkiye, CHE per capita was positively associated with DALY rates (β = +0.18, p = 0.041; Table-I). Given the ecological time-series design, this finding should be interpreted as correlational and may reflect reverse causality, whereby a growing diabetes burden increases expenditure needs.^13^,^16^,^17^

**Table-I:** Associations Between Health Expenditure and Diabetes Burden (H2a-H2b).

Country / Model	Period	β	SE	p	R²	N	Interpretation
OECD (Panel FE)	2010-2021	-0.32	0.08	<0.001	0.41	418	Higher CHE associated with lower DALY
Türkiye (Time Series)	2010-2021	+0.18	0.07	0.041*	0.29	12	Rising CHE coincides with higher DALY

***Note:*** DALY refers to diabetes-related rates per 100,000 population. CHE per capita is reported in US$ PPP. Both DALY and CHE per capita were log-transformed in the H2 models. p < 0.05 significant.

### H3a-H3b (Financing structure and equity):

For 2010-2021, Türkiye’s mean out-of-pocket spending (OOPS) ratio was 16.7%. A positive association was found between OOPS and DALY (β = 0.042, p = 0.048; Table-II), confirming that higher out-of-pocket costs relate to a greater disease burden. Conversely, OECD countries with a higher public share of expenditure (GGHE-D) reported significantly lower OOPS (β = -0.74, p < 0.001; Table-II), supporting the protective role of public financing.^18^

**Table-II:** Financing Structure and Equity Indicators (H3a-H3b).

Country / Model	Relationship	β	SE	p	R² / N	Interpretation
Türkiye (H3a)	OOPS (%CHE) → DALY	+0.042	0.018	0.048*	R² = 0.36, N=12	Higher OOPS linked with greater DALY
OECD (H3b)	GGHE-D (%CHE) → OOPS	-0.74	0.09	<0.001	N=420	Greater public spending reduces OOPS

p < 0.05 significant.

Türkiye’s diabetes-related DALYs and avoidable admissions remained higher than OECD levels (H1a-H1b), underscoring the need for stronger prevention and integrated care. YLD correlated positively with admissions (H1c), linking morbidity to service use. Across the OECD, higher spending related to lower burden, but in Türkiye, rising costs aligned with higher DALYs (H2a-H2b). Greater out-of-pocket spending (OOPS) increased burden, while higher public financing reduced OOPS (H3a-H3b). These results stress the need for financial protection and a value-based health care approach focused on prevention, outcomes, and sustainability.^13^,^14^,^17^,^18^

## DISCUSSION

The study highlights persistent gaps between Türkiye and OECD countries in diabetes outcomes and spending efficiency, providing new evidence of a ‘spending-outcome gap’ in a middle-income setting. Health financing and reimbursement policies influence hospital utilisation patterns; in Türkiye, limited primary-care reimbursement for diabetes medications under the Social Security Institution (SGK) may lead patients to seek hospital-based prescriptions, increasing admissions independent of clinical need. Addressing such structural factors is essential for accurate data interpretation and effective value-based health reforms.

Across OECD countries, higher per-capita spending was linked to lower DALYs, while in Türkiye, rising expenditures coincided with poorer outcomes. This correlation is non-causal, as the data are descriptive and ecological. The pattern may also reflect reverse causality, where a growing disease burden increases spending needs rather than signalling inefficiency. These findings underscore that the allocation and prioritization of resources, particularly the balance between preventive and treatment-oriented spending, may be critical for improving value generation. As emphasized in the literature, value depends not on the absolute level of spending but on its ability to produce measurable patient-centred outcomes.^13^,^14^,^18^

Financing structures significantly influence outcomes. In Türkiye, higher out-of-pocket spending (OOPS) correlated with a greater diabetes burden, while OECD countries with higher public spending (GGHE-D) showed lower OOPS. Strengthening financial protection could enhance equity and sustainability. Evidence from Turkish heart-failure patients also show that chronic diseases create major system costs, highlighting the value of cost-of-illness analyses.^19^

Preventive and digital health strategies offer key opportunities to reduce the diabetes burden. National programs such as e-Nabız and the Türkiye Diabetes Program show how digital tools and lifestyle interventions support prevention.^3^ Studies on wearables and transition-readiness tools demonstrate integration of patient-reported outcomes into chronic care.^6^ Regionally, DALY-based evidence links low physical activity to much of the MENA diabetes burden, underscoring the value of prevention.^20^

### Limitations:

The time-series analysis spans twelve years (2010-2021) with comparable OECD and WHO data, limiting statistical power and causal inference. Data on avoidable diabetes admissions in Türkiye (2015-2017) further constrain analyses. The ecological design and aggregate NHA data restrict detailed evaluation of preventive or chronic-care spending. Despite these limits, the study offers valuable insights for value-based policy. Reliable outcome measurement remains central to VBHC, promoting innovation, efficiency, and system sustainability.

## CONCLUSION

This study found that Türkiye had higher diabetes-related DALY rates and avoidable diabetes hospital admissions than the OECD average during 2010-2021. Across OECD countries, higher CHE per capita was associated with lower DALY rates, whereas in Türkiye, rising CHE per capita was observed alongside higher DALY rates. OOPS was positively associated with DALY in Türkiye, while GGHE-D was inversely associated with OOPS across OECD countries.

These findings suggest that health spending alone may be insufficient unless linked to prevention, outcome measurement, and financial protection. A macro-level VBHC approach based on a “measure-compare-learn-incentivize” cycle may help Türkiye improve diabetes outcomes and spending efficiency.^13^,^21^ Strengthening primary care and reminder-based preventive interventions may reduce avoidable complications and service use.^22^

Routine use of patient-reported outcome measures can improve transparency in how spending translates into results.^14^ Reducing OOPS through stronger public financing may enhance equity and financial protection.^16^,^18^ Digital tools such as e-Nabız and wearable technologies may further support prevention and continuous outcome monitoring.^3^,^6^,^17^,^23^

### Recommendations:

Future studies should assess how spending on prevention, primary care, and complication management influences the diabetes burden. Micro-level data (e.g., TUIK Health Surveys) can reveal patterns in out-of-pocket costs, access, and inequality. Long-term cost-effectiveness and QALY/DALY models should evaluate key complications. Comparative and digital health studies are also needed to measure program effectiveness and efficiency within the OECD framework.

### Authors’ Contributions:

**IHK:** Conceptualized the study, Literature search, data analysis, and drafted the manuscript.

**DGK:** Literature search, data interpretation, critical revision.

All authors have read and approved the final manuscript and are responsible for the integrity of the study.
